# A Medical Causerie

**Published:** 1916-01-29

**Authors:** 


### A MEDICAL CAUSERIE.

The Medical War Committees, alike in England and in Scotland, have not yet reached their full ambition—namely, the registration of all medical practitioners of military age, which, in the case of doctors, it must be remembered, is not forty-one but forty-five. What the Committees desire is to have all medical men below this age under an engagement to accept a commission in the R.A.M.C. if the demand is made on them. Of course, this does not mean an expectation that all of them, or anything like all of them, will be required. But it does mean that if the Army authorities call for more doctors the selection of those who ought to respond shall be made, not by the individual judgment but by a body which may claim to be fairly representative of the profession. In a word, the doctors are asked to put themselves in a position similar to that occupied by those who have attested, or will soon be deemed by the law to have attested, under Lord Derby's scheme, the tribunal which is to decide the duty of the individual practitioner being a local medical committee in consultation with the central authorities. The scheme of arrangement includes much more than the mere supply of the wants of the Army. It comprises an ordered attempt to meet the needs of the civil population by a plan which will continue the practices of those practitioners who will be absent on military duty; and this will obviously need the co-operation of medical men who are beyond the military age. In this part of the work there are clearly concerned the Local Government Board and the National Health Insurance Commissioners, and these authorities are co-operating with the Medical War Committees in the interests of the civil population.

There are two main hindrances to the success of the above schemes. One is the frequent report that many men who have joined the R.A.M.C. find that they have little or nothing to do, and that in certain districts medical officers are much in excess of the demands of the situation. Of course, it must be remembered that the Army authorities have to be prepared for a maximum demand such as is likely to occur when there is fighting on a large scale, and that just now, when the number of casualties is comparatively small, the doctors must in places be largely unoccupied. At the same time there is evidence that so far the organisation of the R.A.M.C. leaves something to be desired, and that by a judicious distribution of officers a much greater amount of work could be secured from the present *personnel.* So long as the existing state of matters continues it will be difficult to convince many members of the profession that it is really necessary for them to give up their practices for the sake of joining the Army. Another hindrance is the failure to utilise the help of practitioners who are willing to give part-time service in the military hospitals and camps. In the early days of the war men were asked to volunteer for this duty, and many responded readily to the call. Yet in a number of districts nothing further has been heard on the matter, and from this has spread the belief that the Army is not really so urgently in need of doctors as the authorities proclaim. Here, again, it looks as though there were some failure in organisation. Certainly there are many doctors who are willing to give practical and systematic help, but who are quite unable to leave their practices and to give full-time service. It is only natural that, having been asked to volunteer, they should conclude that there is no very great need seeing that their offers have not been utilised.

In some quarters there is displayed a disposition to gird at the British Medical Association for the part it has taken in the organisation of the Central War Committee for England and Wales. It is quite true that the idea of the Committee took origin in the Association, but even from the outset representatives of other interests were added, and the number of these has now been substantially increased, so that it would be difficult to deny the Committee a national character. Had the Association not taken the first step the question may well be raised whether the Committee would ever have come into existence, and in this case there would have been no official channel of communication between the profession and the War Office—at least not in England and Wales. In such circumstances, all the doctors of military age would have come under Lord Derby's scheme or under the new Military Service Bill, and would have had to submit their individual pleas to the local tribunals, composed, as these doubtless will be, of laymen. At present the War Office has been content to leave the whole question of the supply of doctors to the Army to the professional Committees, and only in the event of these failing will other steps be contemplated. In Scotland the Central War Committee took origin with the Edinburgh Colleges and the Glasgow Faculty, and it has secured general confidence as fully representative of the profession. Perhaps it would have been well had a similar action been instituted south of the Tweed. But as a matter of fact no one moved in the matter until last July, and then the British Medical Association took it in hand. It is not necessary to approve all the policies of the Association in order to agree that in this respect its action has been useful both to the profession and to the country. Moreover, it has generously recognised the importance of commanding the co-operation of those who stand outside its own borders, and has provided both offices and funds for the working of the organisation. In these circumstances it is surely somewhat ungracious to claim that the Association in its official capacity ought to be divorced from the War Committee. Such a claim raises the question—How without the co-operation of the Association is the work to be continued? That the work must be continued if the profession is to remain master in its own house is certain. Criticism should be directed to aid, not to hinder the Committee's work.

